# Longitudinal relationship between screen-based sedentary behavior and nutrient intake in Japanese children: an observational epidemiological cohort study

**DOI:** 10.1265/ehpm.23-00307

**Published:** 2024-03-13

**Authors:** Hiromasa Tsujiguchi, Yuriko Sakamoto, Akinori Hara, Keita Suzuki, Sakae Miyagi, Masaharu Nakamura, Chie Takazawa, Kim Oanh Pham, Thao Thi Thu Nguyen, Yasuhiro Kambayashi, Yukari Shimizu, Hirohito Tsuboi, Yasuki Ono, Toshio Hamagishi, Aki Shibata, Koichi Hayashi, Tadashi Konoshita, Hiroyuki Nakamura

**Affiliations:** 1 Department of Hygiene and Public Health, Faculty of Medicine, Institute of Medical, Pharmaceutical and Health Sciences, Kanazawa University, 13-1 Takaramachi, Kanazawa, Ishikawa, Japan; 2 Department of Public Health, Graduate School of Advanced Preventive Medical Sciences, Kanazawa University, 13-1 Takaramachi, Kanazawa, Ishikawa, Japan; 3 Advanced Preventive Medical Sciences Research Center, Kanazawa University, 13-1 Takaramachi, Kanazawa, Ishikawa, Japan; 4 Innovative Clinical Research Center, Kanazawa University, 13-1 Takaramachi, Kanazawa, Ishikawa, Japan; 5 Data Management Department, Asia Center for Air Pollution Research, 1182 Sowa Nishi-ku, Niigata, Japan; 6 Department of Epidemiology, Faculty of Public Health, Haiphong University of Medicine and Pharmacy, 72A Nguyen Binh Khiem, Ngo Quyen (district), Hai Phong, Vietnam; 7 Department of Public Health, Faculty of Veterinary Medicine, Okayama University of Science, 1-3 Ikoi-no-oka, Imabari, Ehime, Japan; 8 Department of Nursing, Faculty of Health Sciences, Komatsu University, 14-1 He Mukai-motoori-machi, Komatsu, Ishikawa, Japan; 9 Graduate School of Human Nursing, The University of Shiga Prefecture, 2500 Hassaka-cho, Hikone, Shiga, Japan; 10 Department of Neuropsychiatry, Graduate School of Medicine, Hirosaki University, 1 Bunkyocho, Hirosaki, Aomori, Japan; 11 Department of Nursing and Rehabilitation, Chubu Gakuin University, 2-1 Kirigaoka, Seki, Gifu, Japan; 12 Department of Food Sciences and Nutrition, School of Human Environmental Sciences, Mukogawa Women’s University, 6-46 Ikebirakicho, Nishinomiya, Hyogo, Japan; 13 Third Department of Internal Medicine, Faculty of Medical Sciences, University of Fukui, 23-3 Matsuokashimoaiduki, Eiheiji, Fukui, Japan

**Keywords:** Screen, Television, Personal computer, Mobile phone, Nutrients, Children, Longitudinal

## Abstract

**Background:**

Concerns regarding the impact of screen-based sedentary behavior on health have been increasing. Therefore, the present study investigated the longitudinal relationship between multiple screen time and nutrient intake in children and adolescents.

**Methods:**

The present study was conducted utilizing 3 years longitudinal data. Study subjects were 740 Japanese children aged between 6 and 12 years at baseline and between 9 and 15 years in the follow-up. Screen-based sedentary behavior was assessed using screen time, including television (TV) viewing, personal computer (PC) use, and mobile phone (MP) use. The main outcomes were the intakes of nutrients. Mixed effect multivariate linear regression analyses were used to examine the longitudinal relationship between screen-based sedentary time and nutrient intake. Covariates included in the multivariable analysis consisted of sex, age, solitary eating, skipping breakfast, staying up late, and body weight status, as confounders, and physical inactivity, as mediator.

**Results:**

In boys, a longer total screen time longitudinally correlated with higher intake of energy and lower intakes of protein, dietary fiber, minerals, and vitamins. In girls, longer total screen time longitudinally associated with higher intake of sucrose and lower intakes of protein, minerals, and vitamins. In boys, a longer TV viewing time was associated with higher intake of sucrose and lower intakes of protein, minerals, and vitamins. In girls, a longer TV viewing time was associated with higher intake of carbohydrates and lower intakes of protein, fat, minerals, and vitamins. In boys, relationships were observed between a longer PC use time and higher intakes of energy as well as lower intakes of protein, minerals, and vitamins. Relationship was observed between longer PC use time and lower intakes of minerals in girls. An increased MP use time was associated with higher intakes of energy, and lower intakes of protein, sucrose, dietary fiber, minerals, and vitamins in boys. A longer MP use time was associated with higher intakes of fat, and salt as well as lower intakes of carbohydrates, protein, minerals, and vitamins in girls.

**Conclusions:**

The present results revealed that longer screen-based sedentary behaviors were longitudinally associated with nutrient intake in children and adolescents. Future study is needed to elucidate these relationships.

**Supplementary information:**

The online version contains supplementary material available at https://doi.org/10.1265/ehpm.23-00307.

## Introduction

Screen time refers to a prolonged viewing time of various screen types, such as televisions (TV), personal computers (PC), and mobile phones (MP). Screen time is theoretically regarded as a sedentary behavior, which is characterized by the time spent in activities with an energy expenditure of less than 1.5 metabolic equivalent of task (METs) in a seated, reclined, or recumbent posture [1].

Global screen time guidelines recommend limiting screen time to ≤2 hours per day for children [2]. However, the majority of children, approximately 40 to 80%, do not meet screen time recommendations [3].

Screen time significantly contributes to overall sedentariness [4] and reduces energy expenditure [5]. Screen-based sedentary behavior has been suggested to contribute to the development of health issues that may persist into adulthood, including excess weight [6]. A recent systematic review concluded that there is moderately strong evidence for the relationship between screen time and obesity [7].

Screen-based sedentary behavior may be associated with obesity mainly due to the effects of an unhealthy diet [8]. A recent study indicated a strong relationship between screen-based sedentary behavior and unhealthy diet [7]. Exposure to food advertisements, interference with the physiological signs of satiety, or increased levels of stress might intermediate such relationships. However, the relationship between screen-based sedentary behavior and nutrient intake has not yet been examined in detail, particularly in children. Furthermore, the majority of research on screen-based sedentary behavior and diet has mainly focused on TV viewing [7]. Although TV viewing remains a popular form of screen-based sedentary behavior for children, the use of PC and MP has become a part of everyday life with technological advances. The impact of each screen on diet might be different because each screen might include different types of advertisements, interference to the physiological signs, or levels of stress. However, data on other forms of screen time besides TV are limited, reflecting the lack of evidence on the effects of new screen time patterns on children’s nutrient intake [7]. A few studies have examined PC use and even fewer have investigated MP [7]. Therefore, the extent to which the above findings may be generalized to more modern forms of screen use remains unclear. Moreover, a limitation shared by all studies is their cross-sectional design. Longitudinal studies are important for confirming causality rather than associations.

Therefore, the present study examined the longitudinal relationships between multiple screen-based behaviors (TV viewing, PC use, and MP use) and nutrient intake in a sample of children in Japan. By clarifying the present aim of this study, it would be beneficial to elucidate which type of screen-based sedentary behavior should be focused for intervention programs. To the best of our knowledge, this relationship has not yet been examined in detail.

## Methods

### Data and participants

This was an observational, epidemiological, and longitudinal study. We utilized data from the Shika study. The Shika study is an ongoing population-based survey that aims to investigate the lifestyle and health status of the Japanese population. Shika town is located in a rural area of Ishikawa prefecture, Japan, with a population of 20,422 individuals (Statistics Bureau of Japan, 2016).

This longitudinal study was primarily designed to assess nutrient intake and associated behaviors in a sample of Japanese children. We collected 3-year interval data for this analysis between October and December at two time points in 2013 (baseline) and 2016 (follow-up). The periods during baseline and follow-up were same for all the children. Linked dataset was constructed in 2020. Exposure and outcome variables were measured both at baseline and in the follow-up. Data on screen-based sedentary behavior and dietary intake were collected via proxy-administered questionnaires. Data were collected from the guardians of children aged between 6 and 12 years old at baseline and 9 and 15 years in the follow-up. All students of compulsory school age who went to elementary schools in the town at baseline were eligible to participate in the study. We distributed questionnaires in collaboration with these schools. We asked the guardians to fill in the questionnaire with the assistance of their children, as much as possible, in order to accurately reflect their children’s general behavior to the greatest extent possible.

**Fig. 1:**
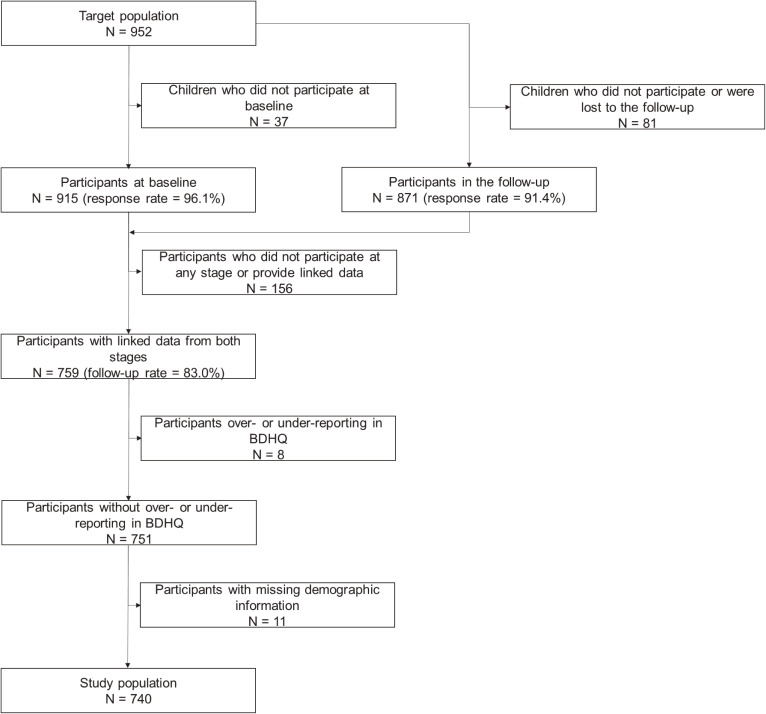
Inclusion criteria

At baseline, 952 children were invited and 915 ultimately participated (response rate = 96.1%). In the follow-up, the same children were invited again and 871 children participated (response rate = 91.4%). We combined the data of 759 children who participated at both time points and provided sufficiently linked data (follow-up rate = 83.0%). Among the 759 participants with linked data, 8 were considered to be under- or over-reporters on energy intake. Therefore, they were excluded from analyses. Among the remaining 751 participants, 11 had data missing on demographic variables and were excluded. Therefore, 740 participants were ultimately analyzed as study subjects. The flow of inclusion is shown in Fig. 1.

Participation in this study was voluntary. None of the participants received any remuneration. Patients or the public were not involved in the conceptualization, design, planning, or carrying out of the study. The present study was conducted in accordance with the Declaration of Helsinki, and the protocol was approved by the Ethics Committee of Kanazawa University (No. 2568). Written informed consent was obtained from the guardians of all participants prior to data collection. A detailed description on the methodology has been provided in a previous study [10, 11].

### Primary measures

#### Screen-based sedentary time (independent variable)

Screen-based sedentary times were assessed based on a questionnaire on TV viewing, PC use, and MP use both at baseline and in the follow-up. These items assessed the mean time spent on each screen on a typical day through specific questions for each device. This type of instrument has been used in other epidemiological studies, and has been reported to have acceptable validity and reliability for quantifying screen time [12]. Total screen time was the sum of time spent on TV, PC, and MP. According to the recent recommendations by the American Academy of Pediatrics (AAP), no more than 2 h daily of sedentary screen time is recommended for children and adolescents [2]. Screen time was categorized as moderate and higher according to the cut-off point of ≥2 hours/day for total screen time and ≥1 hour/day for each screen time.

#### Nutrient intake (dependent variables)

The main outcome was the intake of nutrients extracted from a validated food frequency questionnaire. We utilized the brief self-administered dietary history questionnaire (BDHQ) both at baseline and in the follow-up. BDHQ asks about dietary history during the preceding month. It is based on a comprehensive version of a validated self-administered dietary history questionnaire (i.e., DHQ) [13]. They were previously shown to have acceptable validity [14, 15]. Reported food and beverage intakes were converted into energy, macronutrient, and micronutrient values using a computer algorithm for BDHQ. Nutrient intake was reported in terms of energy density (% energy for macronutrients and per 1,000 kcal for micronutrients). Details on BDHQ are described elsewhere [16]. Participants who reported less than 600 kcal or more than 4,000 kcal energy intake per day were excluded from analyses as they were considered to be under- or over-reporters.

#### Baseline characteristics (covariates)

Covariates included in the multivariable analysis consisted of sex, age, solitary eating, skipping breakfast, staying up late, and body weight status, as confounders. Physical inactivity was also included as a mediator [17, 18]. Body weight status was based on the Rohrer index computed from self-reported heights and weights. The Rohrer index was calculated based on the standard formula (kg/m^3^ × 10). Although participation in screen-based sedentary behaviors and physical inactivity might occur independently, research suggests that both types of behaviors may be associated with dietary behavior [19]. The questionnaire was used to assess the level of physical inactivity, which was evaluated by asking the time spent doing physical activity on an average day.

### Statistical analysis

The distribution of the data was assumed to be normal from central limit theorem [20], because the sample size was more than 100. Descriptive statistics were used to describe the baseline characteristics, screen-based sedentary behavior, and nutrient intake of the study subjects. Mean (M) and standard deviation (SD) values were summarized for quantitative variables, and frequencies (N) and percentages (%) for qualitative variables. Paired *t*-tests were used to examine the significance of differences in screen time and nutrient intake between baseline and the follow-up. Mixed-effects multivariate linear regression analyses, adjusting for sex, age, solitary eating, skipping breakfast, staying up late, body weight status, and physical inactivity, were used to provide parameter estimates (β) and 95% confidence intervals (CI) for the longitudinal relationship between each screen time and nutrient intake across baseline to the follow-up. Forced entry method was applied to use all the potential variables, which might affect both screen-based sedentary behavior and diet, for adjustment. Statistical Package for Social Science version 27 was used for these analyses. Statistical tests were two-sided at a significance level of 5%.

## Results

### Characteristics of study subjects

The characteristics of the studied subjects are shown in Table 1. A total of 740 participants provided data for the variables of interest and were included in analyses. Boys accounted for 48·5% (N = 359) of subjects and girls 51.5% (N = 381). The mean age of subjects was 9·19 (SD = 1.78) years. The percentages of subjects with a longer total screen, TV viewing, PC use, and MP use were 26·4, 84·2, 5·6, and 3·0% at baseline and 61·7, 85·1, 19·5, and 24·7% in the follow-up, respectively, showing a significant difference in total screen, PC use, and MP use. The mean intake of energy was significantly higher in the follow-up than at baseline. The intakes of protein, sucrose, dietary fiber, potassium, iron, copper, manganese, retinol equivalent, beta-carotene equivalent, vitamin B1, vitamin B2, niacin, vitamin B6, vitamin B12, vitamin C, vitamin D, alpha-tocopherol, vitamin K, folic acid, and pantothenic acid were significantly higher at baseline than in the follow-up.

**Table 1:** Characteristics (N = 740)

	**Baseline**		**Follow-up**		**p-value**
	**Mean/number**	**SD/%**	**Mean/number**	**SD/%**	
Sex	359	48.5%	-	-	-
Age	9.19	1.78	-	-	-
Solitary eating	53	7.16%	-	-	-
Physical inactivity	173	23.4%	-	-	-
Skipping breakfast	50	6.8%	-	-	-
Staying up late	196	26.5%	-	-	-
The Rohrer index	129.60	19.04	-	-	-
Longer total screen time	195	26.4%	457	61.7%	<0.001
Longer TV viewing time	623	84.2%	630	85.1%	0.675
Longer PC using time	41	5.6%	145	19.5%	<0.001
Longer MP using time	22	3.0%	183	24.7%	<0.001
Energy (kcal)	1674.20	531.12	1790.09	673.07	<0.001
Protein (%E)	14.03	2.00	13.79	2.07	0.005
Fat (%E)	28.42	4.72	28.52	4.67	0.669
Carbohydrates (%E)	56.13	5.81	56.14	5.71	0.975
Sucrose (%E)	2.40	1.41	2.19	1.25	<0.001
Total dietary fiber (g/1000 kcal)	5.80	1.18	5.59	1.24	<0.001
Salt equivalent (g/1000 kcal)	6.13	1.51	6.22	1.43	0.063
Potassium (mg/1000 kcal)	1218.37	248.72	1139.80	243.74	<0.001
Calcium (mg/1000 kcal)	349.61	82.39	347.42	96.69	0.575
Magnesium (mg/1000 kcal)	122.35	18.31	121.36	19.20	0.182
Phosphorus (mg/1000 kcal)	563.32	85.26	558.80	95.23	0.239
Iron (mg/1000 kcal)	3.75	0.65	3.64	0.71	<0.001
Zinc (mg/1000 kcal)	4.43	0.46	4.42	0.50	0.553
Copper (mg/1000 kcal)	0.61	0.08	0.60	0.08	0.004
Manganese (mg/1000 kcal)	1.65	0.40	1.57	0.41	<0.001
Retinol equivalent (µg/1000 kcal)	297.54	136.08	266.23	149.04	<0.001
Beta carotene equivalent (µg/1000 kcal)	1593.91	747.73	1357.32	777.05	<0.001
Vitamin B1 (mg/1000 kcal)	0.40	0.07	0.39	0.07	<0.001
Vitamin B2 (mg/1000 kcal)	0.70	0.15	0.67	0.16	<0.001
Niacin (mg/1000 kcal)	6.53	1.57	6.32	1.55	0.002
Vitamin B6 (mg/1000 kcal)	0.56	0.12	0.53	0.12	<0.001
Vitamin B12 (µg/1000 kcal)	3.54	1.60	3.22	1.52	<0.001
Vitamin C (mg/1000 kcal)	53.98	21.40	47.66	19.90	<0.001
Vitamin D (µg/1000 kcal)	4.85	2.49	4.38	2.55	<0.001
α-tocopherol (mg/1000 kcal)	3.56	0.73	3.46	0.73	0.002
Vitamin K (µg/1000 kcal)	111.72	47.12	106.62	51.29	0.016
Folic acid (µg/1000 kcal)	149.87	40.43	139.56	40.77	<0.001
Pantothenic acid (mg/1000 kcal)	3.35	0.55	3.23	0.56	<0.001

### Longitudinal relationship between total screen time and nutrient intake

The longitudinal relationships between total screen time and nutrient intake are shown in Tables 2 and 3.

**Table 2:** Longitudinal relationship between total screen time and nutrient intake in boys (N = 359)

**Parameter**	**Estimated ** **value**	**Standard ** **error**	**95% confidence interval**		**Standerdized ** **estimated value**	**p-value**
			**Lower**	**Upper**		
Energy (kcal)	86.607	38.520	10.965	162.249	0.072	0.025
Protein (%E)	−0.447	0.136	−0.714	−0.181	−0.118	0.001
Fat (%E)	−0.042	0.345	−0.720	0.636	−0.005	0.903
Carbohydrates (%E)	0.446	0.417	−0.373	1.265	0.041	0.285
Sucrose (%E)	−0.123	0.089	−0.298	0.052	−0.050	0.169
Total dietary fiber (g/1000 kcal)	−0.194	0.083	−0.357	−0.031	−0.081	0.019
Salt equivalent (g/1000 kcal)	−0.052	0.083	−0.214	0.111	−0.019	0.532
Potassium (mg/1000 kcal)	−62.655	16.975	−95.989	−29.321	−0.129	<0.001
Calcium (mg/1000 kcal)	−19.398	6.445	−32.056	−6.741	−0.108	0.003
Magnesium (mg/1000 kcal)	−2.922	1.222	−5.323	−0.521	−0.082	0.017
Phosphorus (mg/1000 kcal)	−21.893	6.149	−33.969	−9.817	−0.126	<0.001
Iron (mg/1000 kcal)	−0.079	0.047	−0.171	0.014	−0.060	0.095
Zinc (mg/1000 kcal)	−0.088	0.031	−0.150	−0.026	−0.097	0.005
Copper (mg/1000 kcal)	−0.009	0.005	−0.018	0.000	−0.062	0.056
Manganese (mg/1000 kcal)	−0.046	0.030	−0.104	0.012	−0.058	0.117
Retinol equivalent (µg/1000 kcal)	−10.258	11.803	−33.433	12.917	−0.032	0.385
Beta carotene equivalent (µg/1000 kcal)	−120.166	52.691	−223.651	−16.681	−0.077	0.023
Vitamin B1 (mg/1000 kcal)	−0.015	0.005	−0.024	−0.006	−0.111	0.002
Vitamin B2 (mg/1000 kcal)	−0.040	0.010	−0.061	−0.020	−0.132	<0.001
Niacin (mg/1000 kcal)	−0.190	0.105	−0.396	0.015	−0.064	0.069
Vitamin B6 (mg/1000 kcal)	−0.026	0.008	−0.041	−0.011	−0.115	0.001
Vitamin B12 (µg/1000 kcal)	−0.223	0.105	−0.430	−0.016	−0.078	0.035
Vitamin C (mg/1000 kcal)	−3.963	1.410	−6.731	−1.194	−0.097	0.005
Vitamin D (µg/1000 kcal)	−0.476	0.173	−0.815	−0.137	−0.101	0.006
α-tocopherol (mg/1000 kcal)	−0.022	0.054	−0.128	0.084	−0.015	0.682
Vitamin K (µg/1000 kcal)	−3.507	3.340	−10.066	3.053	−0.037	0.294
Folic acid (µg/1000 kcal)	−6.728	2.740	−12.109	−1.348	−0.083	0.014
Pantothenic acid (mg/1000 kcal)	−0.145	0.036	−0.216	−0.075	−0.132	<0.001

Mixed-effects multivariable linear regression analyses, adjusting for sex, age, solitary eating, skipping breakfast, staying up late, body weight status, and physical inactivity.

**Table 3:** Longitudinal relationship between total screen time and nutrient intake in girls (N = 381)

**Parameter**	**Estimated ** **value**	**Standard ** **error**	**95% confidence interval**		**Standerdized ** **estimated value**	**p-value**
			**Lower**	**Upper**		
Energy (kcal)	−64.257	33.561	−130.151	1.636	−0.065	0.056
Protein (%E)	−0.382	0.148	−0.672	−0.092	−0.091	0.010
Fat (%E)	0.008	0.346	−0.671	0.687	0.001	0.982
Carbohydrates (%E)	0.394	0.423	−0.437	1.224	0.035	0.352
Sucrose (%E)	0.211	0.103	0.009	0.414	0.075	0.041
Total dietary fiber (g/1000 kcal)	−0.104	0.087	−0.275	0.067	−0.043	0.232
Salt equivalent (g/1000 kcal)	0.004	0.091	−0.176	0.184	0.001	0.965
Potassium (mg/1000 kcal)	−46.377	17.493	−80.723	−12.031	−0.091	0.008
Calcium (mg/1000 kcal)	−2.922	1.222	−5.323	−0.521	−0.103	0.017
Magnesium (mg/1000 kcal)	−3.747	1.311	−6.321	−1.174	−0.098	0.004
Phosphorus (mg/1000 kcal)	−18.741	6.575	−31.652	−5.830	−0.101	0.005
Iron (mg/1000 kcal)	−0.091	0.049	−0.187	0.005	−0.066	0.063
Zinc (mg/1000 kcal)	−0.091	0.034	−0.157	−0.024	−0.092	0.008
Copper (mg/1000 kcal)	−0.017	0.005	−0.028	−0.006	−0.105	0.002
Manganese (mg/1000 kcal)	0.004	0.029	−0.053	0.062	0.005	0.884
Retinol equivalent (µg/1000 kcal)	−22.051	9.253	−40.216	−3.885	−0.085	0.017
Beta carotene equivalent (µg/1000 kcal)	−62.213	53.097	−166.467	42.040	−0.040	0.242
Vitamin B1 (mg/1000 kcal)	−0.006	0.005	−0.016	0.003	−0.042	0.210
Vitamin B2 (mg/1000 kcal)	−0.028	0.011	−0.049	−0.007	−0.089	0.010
Niacin (mg/1000 kcal)	−0.036	0.112	−0.256	0.183	−0.011	0.746
Vitamin B6 (mg/1000 kcal)	−0.013	0.008	−0.029	0.003	−0.052	0.122
Vitamin B12 (µg/1000 kcal)	−0.170	0.118	−0.402	0.062	−0.051	0.151
Vitamin C (mg/1000 kcal)	−0.320	1.467	−3.200	2.560	−0.008	0.827
Vitamin D (µg/1000 kcal)	−0.274	0.191	−0.649	0.101	−0.051	0.151
α-tocopherol (mg/1000 kcal)	0.000	0.053	−0.105	0.105	0.000	0.998
Vitamin K (µg/1000 kcal)	−7.357	3.486	−14.202	−0.513	−0.076	0.035
Folic acid (µg/1000 kcal)	−4.695	2.732	−10.060	0.669	−0.058	0.086
Pantothenic acid (mg/1000 kcal)	−0.113	0.037	−0.186	−0.041	−0.101	0.002

Mixed-effects multivariable linear regression analyses, adjusting for sex, age, solitary eating, skipping breakfast, staying up late, body weight status, and physical inactivity.

After accounting for sex, age, solitary eating, skipping breakfast, staying up late, body weight status, and physical inactivity, a longer total screen time longitudinally correlated with higher intake of energy (p = 0.025) and lower intakes of protein (p = 0.001), dietary fiber (p = 0.019), potassium (p < 0.001), calcium (p = 0.003), magnesium (p = 0.017), phosphorus (p < 0.001), zinc (p = 0.005), beta carotene equivalent (p = 0.023), vitamin B1 (p = 0.002), vitamin B2 (p < 0.001), vitamin B6 (p = 0.001), vitamin B12 (p = 0.035), vitamin C (p = 0.005), vitamin D (p = 0.006), folic acid (p = 0.014), and pantothenic acid (p < 0.001) in boys. In girls, a longer total screen time longitudinally correlated with higher intake of sucrose (p = 0.041) and lower intakes of protein (p = 0.010), potassium (p = 0.008), calcium (p = 0.017), magnesium (p = 0.004), phosphorus (p = 0.005), zinc (p = 0.008), copper (p = 0.002), retinol equivalent (p = 0.017), vitamin B2 (p = 0.010), vitamin K (p = 0.035), and pantothenic acid.

### Longitudinal relationship between TV time and nutrient intake

The longitudinal relationships between TV time and nutrient intake are shown in Tables 4 and 5.

**Table 4:** Longitudinal relationship between total screen time and nutrient intake in boys (N = 359)

**Parameter**	**Estimated ** **value**	**Standard ** **error**	**95% confidence interval**		**Standerdized ** **estimated value**	**p-value**
			**Lower**	**Upper**		
Energy (kcal)	−40.795	54.327	−147.459	65.869	−0.025	0.453
Protein (%E)	−0.476	0.191	−0.851	−0.100	−0.091	0.013
Fat (%E)	0.362	0.473	−0.567	1.292	0.029	0.445
Carbohydrates (%E)	0.295	0.573	−0.829	1.420	0.020	0.606
Sucrose (%E)	0.316	0.126	0.067	0.564	0.093	0.013
Total dietary fiber (g/1000 kcal)	−0.155	0.120	−0.390	0.080	−0.047	0.196
Salt equivalent (g/1000 kcal)	0.013	0.119	−0.220	0.246	0.004	0.911
Potassium (mg/1000 kcal)	−30.516	24.160	−77.952	16.920	−0.046	0.207
Calcium (mg/1000 kcal)	−18.434	9.166	−36.430	−0.438	−0.075	0.045
Magnesium (mg/1000 kcal)	−3.861	1.761	−7.318	−0.404	−0.079	0.029
Phosphorus (mg/1000 kcal)	−28.203	8.705	−45.294	−11.112	−0.118	0.001
Iron (mg/1000 kcal)	−0.087	0.067	−0.218	0.044	−0.048	0.195
Zinc (mg/1000 kcal)	−0.127	0.045	−0.215	−0.038	−0.101	0.005
Copper (mg/1000 kcal)	−0.015	0.007	−0.028	−0.001	−0.074	0.032
Manganese (mg/1000 kcal)	−0.052	0.041	−0.133	0.029	−0.047	0.208
Retinol equivalent (µg/1000 kcal)	−25.935	16.241	−57.823	5.952	−0.059	0.111
Beta carotene equivalent (µg/1000 kcal)	−120.977	76.059	−270.312	28.359	−0.056	0.112
Vitamin B1 (mg/1000 kcal)	−0.004	0.007	−0.017	0.009	−0.023	0.531
Vitamin B2 (mg/1000 kcal)	−0.024	0.015	−0.053	0.005	−0.057	0.107
Niacin (mg/1000 kcal)	−0.215	0.150	−0.509	0.079	−0.052	0.151
Vitamin B6 (mg/1000 kcal)	−0.017	0.011	−0.039	0.004	−0.056	0.118
Vitamin B12 (µg/1000 kcal)	−0.407	0.146	−0.694	−0.119	−0.103	0.006
Vitamin C (mg/1000 kcal)	−0.860	2.014	−4.814	3.094	−0.015	0.669
Vitamin D (µg/1000 kcal)	−0.695	0.240	−1.166	−0.224	−0.107	0.004
α-tocopherol (mg/1000 kcal)	−0.036	0.075	−0.184	0.112	−0.018	0.635
Vitamin K (µg/1000 kcal)	−8.068	4.785	−17.463	1.326	−0.061	0.092
Folic acid (µg/1000 kcal)	−7.541	3.936	−15.269	0.186	−0.067	0.056
Pantothenic acid (mg/1000 kcal)	−0.082	0.052	−0.183	0.020	−0.053	0.116

Mixed-effects multivariable linear regression analyses, adjusting for sex, age, solitary eating, skipping breakfast, staying up late, body weight status, and physical inactivity.

**Table 5:** Longitudinal relationship between total screen time and nutrient intake in girls (N = 381)

**Parameter**	**Estimated ** **value**	**Standard ** **error**	**95% confidence interval**		**Standerdized ** **estimated value**	**p-value**
			**Lower**	**Upper**		
Energy (kcal)	−70.769	46.251	−161.566	20.029	−0.052	0.126
Protein (%E)	−0.775	0.204	−1.175	−0.375	−0.133	<0.001
Fat (%E)	−1.096	0.467	−2.012	−0.180	−0.086	0.019
Carbohydrates (%E)	1.958	0.572	0.836	3.081	0.125	0.001
Sucrose (%E)	0.245	0.141	−0.032	0.522	0.063	0.083
Total dietary fiber (g/1000 kcal)	−0.196	0.120	−0.432	0.039	−0.060	0.102
Salt equivalent (g/1000 kcal)	−0.255	0.127	−0.505	−0.005	−0.064	0.045
Potassium (mg/1000 kcal)	−49.556	24.228	−97.119	−1.992	−0.071	0.041
Calcium (mg/1000 kcal)	−16.704	8.993	−34.358	0.950	−0.068	0.064
Magnesium (mg/1000 kcal)	−6.106	1.832	−9.703	−2.509	−0.116	0.001
Phosphorus (mg/1000 kcal)	−29.581	9.097	−47.439	−11.723	−0.116	0.001
Iron (mg/1000 kcal)	−0.194	0.068	−0.327	−0.060	−0.102	0.004
Zinc (mg/1000 kcal)	−0.149	0.047	−0.242	−0.056	−0.110	0.002
Copper (mg/1000 kcal)	−0.013	0.008	−0.028	0.001	−0.060	0.077
Manganese (mg/1000 kcal)	−0.002	0.041	−0.082	0.078	−0.002	0.963
Retinol equivalent (µg/1000 kcal)	3.548	12.663	−21.312	28.407	0.010	0.779
Beta carotene equivalent (µg/1000 kcal)	34.445	73.646	−110.135	179.025	0.016	0.640
Vitamin B1 (mg/1000 kcal)	−0.017	0.007	−0.030	−0.003	−0.083	0.014
Vitamin B2 (mg/1000 kcal)	−0.030	0.015	−0.059	0.000	−0.069	0.047
Niacin (mg/1000 kcal)	−0.570	0.154	−0.872	−0.268	−0.128	0.000
Vitamin B6 (mg/1000 kcal)	−0.036	0.012	−0.058	−0.013	−0.104	0.002
Vitamin B12 (µg/1000 kcal)	−0.295	0.164	−0.616	0.026	−0.065	0.071
Vitamin C (mg/1000 kcal)	−1.530	2.014	−5.484	2.423	−0.026	0.448
Vitamin D (µg/1000 kcal)	−0.663	0.262	−1.178	−0.148	−0.090	0.012
α-tocopherol (mg/1000 kcal)	−0.175	0.073	−0.318	−0.032	−0.086	0.017
Vitamin K (µg/1000 kcal)	−9.473	4.788	−18.873	−0.073	−0.071	0.048
Folic acid (µg/1000 kcal)	−6.076	3.806	−13.549	1.396	−0.055	0.111
Pantothenic acid (mg/1000 kcal)	−0.137	0.051	−0.238	−0.037	−0.089	0.008

Mixed-effects multivariable linear regression analyses, adjusting for sex, age, solitary eating, skipping breakfast, staying up late, body weight status, and physical inactivity.

In boys, a longer TV viewing time was associated with higher intake of sucrose (p = 0.013) and lower intakes of protein (p = 0.013), calcium (p = 0.045), magnesium (p = 0.029), phosphorus (p = 0.001), zinc (p = 0.005), copper (p = 0.032), vitamin B12 (p = 0.006), and vitamin D (p = 0.004). In girls, higher intake of carbohydrates (p = 0.001) and lower intakes of protein (p < 0.001), fat (p = 0.019), salt equivalent (p = 0.045), potassium (p = 0.041), magnesium (p = 0.001), phosphorus (p = 0.001), iron (p = 0.004), zinc (p = 0.002), vitamin B1 (p = 0.014), vitamin B2 (p = 0.047), niacin (p < 0.001), vitamin B6 (p = 0.002), vitamin D (p = 0.012), alpha-tocopherol (p = 0.017), vitamin K (p = 0.048), and pantothenic acid (p = 0.008).

### Longitudinal relationship between PC use time and nutrient intake

The longitudinal relationships between PC use time and nutrient intake are shown in Tables 6 and 7.

**Table 6:** Longitudinal relationship between total screen time and nutrient intake in boys (N = 359)

**Parameter**	**Estimated ** **value**	**Standard ** **error**	**95% confidence interval**		**Standerdized ** **estimated value**	**p-value**
			**Lower**	**Upper**		
Energy (kcal)	136.457	55.036	28.397	244.518	0.080	0.013
Protein (%E)	−0.698	0.193	−1.077	−0.318	−0.130	<0.001
Fat (%E)	0.163	0.488	−0.795	1.120	0.013	0.739
Carbohydrates (%E)	0.480	0.589	−0.677	1.637	0.031	0.416
Sucrose (%E)	0.050	0.128	−0.202	0.302	0.014	0.699
Total dietary fiber (g/1000 kcal)	−0.229	0.120	−0.465	0.006	−0.068	0.056
Salt equivalent (g/1000 kcal)	−0.196	0.119	−0.429	0.038	−0.051	0.100
Potassium (mg/1000 kcal)	−86.100	24.297	−133.806	−38.394	−0.126	<0.001
Calcium (mg/1000 kcal)	−24.080	9.240	−42.223	−5.937	−0.095	0.009
Magnesium (mg/1000 kcal)	−5.992	1.756	−9.440	−2.543	−0.119	0.001
Phosphorus (mg/1000 kcal)	−30.120	8.791	−47.382	−12.859	−0.123	0.001
Iron (mg/1000 kcal)	−0.174	0.067	−0.306	−0.042	−0.095	0.010
Zinc (mg/1000 kcal)	−0.156	0.045	−0.244	−0.067	−0.121	0.001
Copper (mg/1000 kcal)	−0.016	0.007	−0.030	−0.003	−0.078	0.020
Manganese (mg/1000 kcal)	−0.054	0.042	−0.137	0.029	−0.048	0.199
Retinol equivalent (µg/1000 kcal)	−16.115	16.709	−48.922	16.692	−0.036	0.335
Beta carotene equivalent (µg/1000 kcal)	−133.744	76.247	−283.466	15.979	−0.061	0.080
Vitamin B1 (mg/1000 kcal)	−0.020	0.007	−0.034	−0.007	−0.106	0.003
Vitamin B2 (mg/1000 kcal)	−0.044	0.015	−0.073	−0.015	−0.102	0.003
Niacin (mg/1000 kcal)	−0.403	0.150	−0.697	−0.109	−0.096	0.007
Vitamin B6 (mg/1000 kcal)	−0.037	0.011	−0.059	−0.016	−0.117	0.001
Vitamin B12 (µg/1000 kcal)	−0.352	0.150	−0.646	−0.058	−0.087	0.019
Vitamin C (mg/1000 kcal)	−6.474	2.020	−10.440	−2.509	−0.112	0.001
Vitamin D (µg/1000 kcal)	−0.551	0.246	−1.034	−0.069	−0.083	0.025
α-tocopherol (mg/1000 kcal)	−0.083	0.077	−0.234	0.068	−0.041	0.280
Vitamin K (µg/1000 kcal)	−5.115	4.817	−14.573	4.344	−0.038	0.289
Folic acid (µg/1000 kcal)	−9.358	3.950	−17.115	−1.600	−0.081	0.018
Pantothenic acid (mg/1000 kcal)	−0.191	0.052	−0.292	−0.089	−0.122	<0.001

Mixed-effects multivariable linear regression analyses, adjusting for sex, age, solitary eating, skipping breakfast, staying up late, body weight status, and physical inactivity.

**Table 7:** Longitudinal relationship between total screen time and nutrient intake in boys (N = 381)

**Parameter**	**Estimated ** **value**	**Standard ** **error**	**95% confidence interval**		**Standerdized ** **estimated value**	**p-value**
			**Lower**	**Upper**		
Energy (kcal)	12.707	52.498	−90.360	115.774	0.008	0.809
Protein (%E)	−0.391	0.231	−0.844	0.062	−0.058	0.091
Fat (%E)	0.501	0.536	−0.552	1.554	0.034	0.350
Carbohydrates (%E)	−0.183	0.658	−1.474	1.108	−0.010	0.781
Sucrose (%E)	0.200	0.161	−0.116	0.515	0.044	0.215
Total dietary fiber (g/1000 kcal)	−0.082	0.136	−0.349	0.184	−0.022	0.544
Salt equivalent (g/1000 kcal)	−0.093	0.143	−0.374	0.188	−0.020	0.516
Potassium (mg/1000 kcal)	−30.680	27.397	−84.469	23.109	−0.038	0.263
Calcium (mg/1000 kcal)	−19.194	10.201	−39.221	0.833	−0.067	0.060
Magnesium (mg/1000 kcal)	−3.420	2.059	−7.464	0.623	−0.056	0.097
Phosphorus (mg/1000 kcal)	−21.278	10.282	−41.465	−1.091	−0.072	0.039
Iron (mg/1000 kcal)	−0.062	0.077	−0.213	0.088	−0.028	0.416
Zinc (mg/1000 kcal)	−0.125	0.053	−0.229	−0.021	−0.080	0.019
Copper (mg/1000 kcal)	−0.017	0.008	−0.034	0.000	−0.066	0.045
Manganese (mg/1000 kcal)	−0.048	0.046	−0.138	0.042	−0.036	0.297
Retinol equivalent (µg/1000 kcal)	−27.329	14.392	−55.583	0.924	−0.067	0.058
Beta carotene equivalent (µg/1000 kcal)	−72.224	83.025	−235.230	90.782	−0.030	0.385
Vitamin B1 (mg/1000 kcal)	−0.002	0.008	−0.017	0.013	−0.010	0.773
Vitamin B2 (mg/1000 kcal)	−0.031	0.017	−0.064	0.002	−0.062	0.068
Niacin (mg/1000 kcal)	0.058	0.175	−0.286	0.401	0.011	0.741
Vitamin B6 (mg/1000 kcal)	−0.009	0.013	−0.035	0.017	−0.022	0.499
Vitamin B12 (µg/1000 kcal)	−0.179	0.185	−0.542	0.184	−0.034	0.334
Vitamin C (mg/1000 kcal)	0.638	2.287	−3.851	5.128	0.009	0.780
Vitamin D (µg/1000 kcal)	−0.095	0.298	−0.681	0.491	−0.011	0.750
α-tocopherol (mg/1000 kcal)	0.028	0.083	−0.135	0.192	0.012	0.736
Vitamin K (µg/1000 kcal)	−3.532	5.448	−14.228	7.164	−0.023	0.517
Folic acid (µg/1000 kcal)	−5.509	4.277	−13.906	2.888	−0.043	0.198
Pantothenic acid (mg/1000 kcal)	−0.097	0.058	−0.211	0.016	−0.054	0.092

Mixed-effects multivariable linear regression analyses, adjusting for sex, age, solitary eating, skipping breakfast, staying up late, body weight status, and physical inactivity.

In boys, relationships were observed between a longer PC use time and higher intake of energy (p = 0.013) as well as lower intakes of protein (p < 0.001), potassium (p < 0.001), calcium (p = 0.009), magnesium (p = 0.001), phosphorus (p = 0.001), iron (p = 0.010), zinc (p = 0.001), copper (p = 0.020), vitamin B1 (p = 0.003), vitamin B2 (p = 0.003), niacin (p = 0.007), vitamin B6 (p = 0.001), vitamin B12 (p = 0.019), vitamin C (p = 0.001), vitamin D (p = 0.025), folic acid (p = 0.018), and pantothenic acid (p < 0.001). In girls, relationships were observed between a longer PC use time and lower intakes of phosphorus (p = 0.039), zinc (p = 0.019), and copper (p = 0.045).

### Longitudinal relationship between MP use time and nutrient intake

The longitudinal relationships between MP use time and nutrient intake are shown in Tables 8 and 9.

**Table 8:** Longitudinal relationship between total screen time and nutrient intake in girls (N = 359)

**Parameter**	**Estimated ** **value**	**Standard ** **error**	**95% confidence interval**		**Standerdized ** **estimated value**	**p-value**
			**Lower**	**Upper**		
Energy (kcal)	265.874	54.493	158.862	372.886	0.155	<0.001
Protein (%E)	−0.461	0.196	−0.845	−0.076	−0.085	0.019
Fat (%E)	−0.038	0.498	−1.015	0.939	−0.003	0.939
Carbohydrates (%E)	0.317	0.601	−0.864	1.497	0.021	0.599
Sucrose (%E)	−0.331	0.128	−0.582	−0.081	−0.094	0.010
Total dietary fiber (g/1000 kcal)	−0.273	0.119	−0.507	−0.039	−0.080	0.022
Salt equivalent (g/1000 kcal)	0.009	0.119	−0.223	0.242	0.002	0.936
Potassium (mg/1000 kcal)	−92.380	24.388	−140.273	−44.488	−0.134	0.000
Calcium (mg/1000 kcal)	−16.992	9.294	−35.244	1.260	−0.067	0.068
Magnesium (mg/1000 kcal)	−2.587	1.759	−6.041	0.868	−0.051	0.142
Phosphorus (mg/1000 kcal)	−20.092	8.877	−37.524	−2.659	−0.081	0.024
Iron (mg/1000 kcal)	−0.094	0.067	−0.226	0.039	−0.051	0.165
Zinc (mg/1000 kcal)	−0.075	0.045	−0.164	0.014	−0.058	0.098
Copper (mg/1000 kcal)	−0.008	0.007	−0.021	0.005	−0.038	0.245
Manganese (mg/1000 kcal)	−0.065	0.043	−0.149	0.018	−0.057	0.126
Retinol equivalent (µg/1000 kcal)	−27.783	16.989	−61.141	5.574	−0.061	0.102
Beta carotene equivalent (µg/1000 kcal)	−237.555	75.365	−385.577	−89.533	−0.107	0.002
Vitamin B1 (mg/1000 kcal)	−0.022	0.007	−0.035	−0.009	−0.114	0.001
Vitamin B2 (mg/1000 kcal)	−0.041	0.015	−0.070	−0.011	−0.094	0.007
Niacin (mg/1000 kcal)	−0.370	0.150	−0.664	−0.075	−0.087	0.014
Vitamin B6 (mg/1000 kcal)	−0.043	0.011	−0.065	−0.022	−0.134	<0.001
Vitamin B12 (µg/1000 kcal)	−0.391	0.152	−0.689	−0.093	−0.096	0.010
Vitamin C (mg/1000 kcal)	−7.346	2.015	−11.303	−3.388	−0.126	0.000
Vitamin D (µg/1000 kcal)	−0.593	0.249	−1.081	−0.104	−0.088	0.018
α-tocopherol (mg/1000 kcal)	−0.097	0.078	−0.250	0.055	−0.047	0.209
Vitamin K (µg/1000 kcal)	3.138	4.798	−6.286	12.562	0.023	0.513
Folic acid (µg/1000 kcal)	−10.394	3.927	−18.107	−2.681	−0.090	0.008
Pantothenic acid (mg/1000 kcal)	−0.136	0.052	−0.239	−0.034	−0.086	0.009

Mixed-effects multivariable linear regression analyses, adjusting for sex, age, solitary eating, skipping breakfast, staying up late, body weight status, and physical inactivity.

**Table 9:** Longitudinal relationship between total screen time and nutrient intake (N = 381)

**Parameter**	**Estimated ** **value**	**Standard ** **error**	**95% confidence interval**		**Standerdized ** **estimated value**	**p-value**
			**Lower**	**Upper**		
Energy (kcal)	−93.459	48.173	−188.043	1.125	−0.065	0.053
Protein (%E)	0.074	0.212	−0.342	0.490	0.012	0.727
Fat (%E)	1.692	0.493	0.724	2.659	0.127	0.001
Carbohydrates (%E)	−1.750	0.603	−2.935	−0.566	−0.107	0.004
Sucrose (%E)	0.119	0.148	−0.172	0.410	0.029	0.421
Total dietary fiber (g/1000 kcal)	−0.157	0.125	−0.401	0.088	−0.045	0.208
Salt equivalent (g/1000 kcal)	0.363	0.130	0.108	0.619	0.087	0.005
Potassium (mg/1000 kcal)	−55.862	25.026	−105.003	−6.722	−0.076	0.026
Calcium (mg/1000 kcal)	−1.612	9.400	−20.069	16.845	−0.006	0.864
Magnesium (mg/1000 kcal)	−1.182	1.880	−4.874	2.510	−0.021	0.530
Phosphorus (mg/1000 kcal)	3.178	9.441	−15.361	21.717	0.012	0.737
Iron (mg/1000 kcal)	0.002	0.070	−0.136	0.140	0.001	0.975
Zinc (mg/1000 kcal)	−0.037	0.049	−0.133	0.058	−0.026	0.441
Copper (mg/1000 kcal)	−0.021	0.008	−0.036	−0.006	−0.089	0.007
Manganese (mg/1000 kcal)	−0.048	0.042	−0.130	0.034	−0.040	0.252
Retinol equivalent (µg/1000 kcal)	−29.135	13.275	−55.198	−3.072	−0.078	0.029
Beta carotene equivalent (µg/1000 kcal)	−191.828	75.592	−340.258	−43.398	−0.087	0.011
Vitamin B1 (mg/1000 kcal)	−0.009	0.007	−0.022	0.005	−0.042	0.211
Vitamin B2 (mg/1000 kcal)	−0.008	0.015	−0.038	0.022	−0.018	0.603
Niacin (mg/1000 kcal)	0.184	0.160	−0.131	0.498	0.039	0.252
Vitamin B6 (mg/1000 kcal)	−0.012	0.012	−0.036	0.011	−0.034	0.314
Vitamin B12 (µg/1000 kcal)	−0.110	0.169	−0.442	0.223	−0.023	0.517
Vitamin C (mg/1000 kcal)	−4.183	2.094	−8.294	−0.072	−0.069	0.046
Vitamin D (µg/1000 kcal)	0.030	0.274	−0.508	0.568	0.004	0.912
α-tocopherol (mg/1000 kcal)	0.119	0.077	−0.031	0.270	0.056	0.120
Vitamin K (µg/1000 kcal)	0.691	5.014	−9.154	10.536	0.005	0.890
Folic acid (µg/1000 kcal)	−5.900	3.902	−13.562	1.763	−0.051	0.131
Pantothenic acid (mg/1000 kcal)	−0.077	0.053	−0.181	0.027	−0.048	0.145

Mixed-effects multivariable linear regression analyses, adjusting for sex, age, solitary eating, skipping breakfast, staying up late, body weight status, and physical inactivity.

A longer MP use time was associated with higher intakes of energy (p < 0.001) as well as lower intakes of protein (p = 0.019), sucrose (0.010), dietary fiber (p = 0.022), potassium (p = 0.001), phosphorus (p = 0.024), beta carotene equivalent (p = 0.002), vitamin B1 (p = 0.001), vitamin B2 (p = 0.007), niacin (p = 0.014), vitamin B6 (p < 0.001), vitamin B12 (p = 0.010), vitamin C (p < 0.001), vitamin D (p = 0.018), folic acid (p = 0.008), and pantothenic acid (p = 0.009) in boys. A longer MP use time was associated with higher intakes of fat (p = 0.001), and salt equivalent (p = 0.005) as well as lower intakes of carbohydrates (p = 0.004), potassium (p = 0.026), copper (p = 0.007), retinol equivalent (p = 0.029), beta carotene equivalent (p = 0.011), and vitamin C (p = 0.046) in girls.

## Discussion

While many studies have investigated the relationship between screen-based sedentary behavior and diet, few have examined that between screen-based sedentary behavior, including mobile devices, and nutrient intake in children, particularly using longitudinal data. Therefore, the present study investigated the longitudinal relationship between multiple screen times and nutrient intake in children. Although there have been several studies on this topic, to the best of our knowledge, we are the first to assess the longitudinal relationship between screen time, including mobile devices, and nutrient intake.

The main results of the present study revealed that: (1) a longer total screen time longitudinally correlated with higher intakes of energy and sucrose and lower intakes of protein, dietary fiber, minerals, and vitamins; (2) a longer TV viewing time was associated with higher intakes of carbohydrate and sucrose and lower intakes of protein, minerals, and vitamins; (3) a longer PC use time was associated with higher intakes of energy and lower intakes of protein, minerals, and vitamins; (4) a longer MP use time was associated with higher intakes of energy, fat, and salt equivalent and lower intakes of protein, sucrose, dietary fiber, minerals, and vitamins. The present results suggest that all types of screen time, including MP, and nutrient intake do not occur in isolation. Some types of screen-based behaviors may be more deleterious than others, suggesting that different types of screen-based behaviors may differentially impact diet; however, the present results suggest that all types of screen-based behavior affect nutrient intake.

A previous study showed positive association between total screen time and total calorie intake in girls aged 5–11 years [21]. Our study the association in boys. A previous study [22] revealed a relationship between total screen time and a lower fiber intake. Similarly, the present study showed using longitudinal data that boys with a daily screen time of more than two hours in the three devices were less likely to consume dietary fiber than those being exposed to less than two hours. Moreover, the present results demonstrated that a longer overall screen time was longitudinally associated with higher intake of sucrose in girls and lower intakes of protein, minerals, and vitamins in both sex, which was not reported in previous studies.

Although previous studies from the U.S. and European countries found inverse or negative relationships between TV viewing times and carbohydrate intake [23], we observed a positive longitudinal relationship in girls. Specifically, the consumption of sucrose was more likely to be frequent among boys with a longer TV viewing time, which appears to be consistent with previous findings [24]. A relationship has been reported between a longer TV viewing time and lower protein intake [23]. A negative relationship was observed in both sex in the present study using longitudinal data. Although a study on preschoolers or adolescents reported no correlation between TV viewing times and iron intake [25], we noted a negative relationship in girls in the present study. TV viewing was negatively associated with calcium intake in the aforementioned review [26]. Consistent with previous findings, we found that a longer TV viewing time negatively correlated with calcium intake in boys. A study on Spanish adolescents reported no relationship between TV viewing times and magnesium or zinc intake [25], whereas we noted inverse relationships in children. The same study showed that adolescents with longer TV viewing times were less likely to consume vitamin B6 [25]. We obtained the same results in girls, thereby corroborating these findings with longitudinal data. Although the previous study [25] did not indicate relationships between TV viewing times and intakes of vitamin B1, vitamin B2, niacin, vitamin B12, vitamin D, and vitamin E, we revealed longitudinal and positive relationships for vitamin B12 in boys and vitamin B1, vitamin B2, niacin, vitamin D and vitamin E in girls. Furthermore, negative correlations were observed between TV viewing times and the intakes of phosphorus, and copper, in boys, and potassium phosphorus, vitamin K and pantothenic acid in girls.

Limited information is currently available on the relationship between PC use and nutrient intake. Only one study revealed a relationship between longer PC use times and higher energy intake [27]. Similarly, we herein found that boys using PC >1 hour/day consumed more energy than those using PC <1 hour/day. In addition, we noted that protein, potassium, calcium, magnesium, phosphorus, iron, zinc, copper, vitamin B1, vitamin B2, niacin, vitamin B6, vitamin B12, vitamin C, vitamin D, folic acid, and pantothenic acid were less likely to be consumed by boys and phosphorus, zinc, and copper in girls with longer PC use times based on longitudinal data, which was not reported in previous studies.

To the best of our knowledge, this the first study to investigate the relationship between MP use and nutrient intake, particularly using longitudinal data. We revealed that boys with a longer MP use time were more likely to have higher intakes of energy, and lower intakes of protein, sucrose, dietary fiber, potassium, phosphorus, copper, beta carotene equivalent, vitamin B1, vitamin B2, niacin, vitamin B6, vitamin B12, vitamin C, vitamin D, folic acid, and pantothenic acid. In addition, we showed that girls with a longer MP use time were more likely to have higher intakes of fat, salt equivalent and lower intakes of carbohydrates, potassium, copper, retinol equivalent, beta carotene equivalent, and vitamin C in girls.

A systematic review of the relationship between screen time and health outcomes among children and adolescents provided moderately strong evidence for screen time being strongly associated with obesity [7]. Screen-based sedentary behavior may affect the energy balance and, ultimately, the body weight status [28].

Several mechanisms have been proposed to explain the relationship between screen-based sedentary behavior and dietary intake. One of the probable mechanisms by which screen time affects dietary intake is reduced physical activity and an increased intake of obesogenic foods [17]. Physical activity has been proposed as a stress-induced eating repressor, which may eventually limit the consumption of unhealthy food [18]. However, even when physical inactivity was accounted for, relationships between screen-based sedentary behavior and the intakes of various nutrient persisted.

Another explanation is that YouTube, Facebook, or Instagram on screen devices increase exposure to food advertisements, which may promote an intake of high-calorie low-nutrient foods as a possible mediator. Previous studies on advertising and programming content reported the presence of frequent advertisements that promoted the consumption of high levels of energy, sucrose, fats, salt, and other calorie-dense nutrient-poor foods along with a lack of content for low-calorie, nutrient-dense foods, such as fruit and vegetables [8]. Previous studies demonstrated that food and beverage advertisements may increase the consumption of these items among children [29].

Furthermore, screens may also interfere with the physiological signs of satiety and hunger, resulting in unhealthy food choices with the increased consumption of high-calorie, low-nutrient foods [30]. Eating while watching TV has been associated with a longer time spent eating, suggesting that concurrent TV viewing and eating may contribute to overeating (Alblas et al., 2021). Moreover, recent systematic reviews on children and adolescents showed that eating while watching TV was associated with poorer diet quality, including the more frequent consumption of sugar-sweetened beverages and high-fat, high-sugar foods and fewer fruits and vegetables [31].

Screen time may also be associated with increased levels of stress, which may turn into stress-induced eating [32]. The consumption of sweetened food has been suggested to counteract stress by releasing dopamine, which activates pleasurable and rewarding sensations and improves psychological well-being [33].

Substance cravings, such as an excessive sugar intake, are also regarded as an addiction [34] because highly palatable foods activate the same brain regions responsible for pleasure and rewards as drugs [35].

Another possible explanation is that can be drawn about the relationships between personal characteristics, such as the clustering of unhealthy behaviors [36]. An individual who engages in one unhealthy behavior may experience another unhealthy behavior, which may be linked to negative aspects of their personal characteristics [37]. Children with unfavorable lifestyle profiles regarding screen time may be more prone to the consumption of unhealthier foods and beverages.

Socio economic status might be underlying on the association between screen-based sedentary behavior and nutrient intake. Previously, economic status was revealed to be significantly associated with parent-child sedentary behavior in adolescents [38]. Therefore, present results could be explained by differences of economic status. In this regard, education levels might influence on sedentary behavior [39]. Child should be strongly influenced by their parent’s behavior as well. Then educational status and structure of family might affect both children’s sedentary behavior and diet.

The amount or contents of advertisement, effect on physiological signs of satiety, or stress level might differ depend on screen type, which might lead to different results according to screen type. In addition, the type of activity performed in front of a screen may be of key importance for assessing the relationship between screen time and nutrient intake. A recent study on adults showed a positive relationship between PC use times and a healthier food intake, which is in contrast to the present results on children [40]. A possible explanation is that PC use in adults is mostly related to occupational tasks, which leads to mentally active screen-based behaviors [40]. PC use in children is mostly related to recreation, which leads to mentally passive screen-based behaviors. Therefore, PC usage may exert different effects between children and adults. Future studies are needed to elucidate the underlying mechanisms.

The present results are concerning because the nutrient profile combined with screen-based sedentary behavior contributes to the development of obesity, which may persist into adulthood. Although small daily amounts of screen use are not harmful and may have some benefits [7], limiting screen-based screen behavior and promoting healthy eating need to be considered in combination from a public health perspective. In our sample, 26·4% at baseline and 61·7% in the follow-up were high screen users (≥2 hours/day), indicating that screen time increases with aging. Childhood is a crucial period because lifestyle behaviors are modifiable and generally established during this time and are likely to track into adulthood. Therefore, limiting the use of screen devices to 2 hours is strongly recommended for these age groups.

The strength of the present study is that we conducted a complete survey on children in one town. We also collected questionnaires at high response and follow-up rates. These strengths allowed us to examine the longitudinal relationship between screen time and nutrient intake, excluding selection biases, particularly a non-respondent bias or volunteer bias.

The limitations of the present study were as follows. The self-report assessment of screen times, which was not formally validated, may be vulnerable to recall biases, even though self-report methods of quantifying screen time have been shown to have acceptable reliability and validity [12]. Furthermore, despite the use of a validated and detailed one-month food frequency questionnaire, we cannot exclude possible measurement errors in nutrient intake. In defense of this point, adjustments for energy intake were conducted to reduce bias. The data on economic status, which might confound between sedentary behavior and diet, were not gathered in this study. The data on extracurricular activity, commuting time, or sleeping periods which might steel time to take screen-based sedentary behavior and taking unhealthy diet, were not utilized as well. Furthermore, this study was conducted in a rural town in Japan, which needs to be considered when interpreting and making generalizations from the present study.

## Conclusions

The present results revealed that children with longer screen times longitudinally consumed more energy, and less protein, dietary fiber, minerals, and vitamins. Future studies utilizing more detailed data are needed to elucidate these relationships.

## Abbreviations

TV: Television
PC: Personal computer
MP: Mobile phone
METs: Metabolic equivalent of task
WHO: World Health Organization
BDHQ: Brief self-administered dietary history questionnaire
BMI: Body mass index
SD: Standard deviation
CI: Confidence interval

## Supplementary information

**Additional file 1:** **Supplementary table.** Interaction of screen time and sex or age on nutrient intake.

## Declarations

### Availability of data and material

The datasets used and analyzed during this study are not publicly available with the aim of preserving the anonymity of information from participants, but deidentified participant data, study protocol, or informed consent form are available from the corresponding author upon reasonable request after approval of ethical committee.
